# Preoperative Cer(d18:1/24:0) Shows Promise as a Candidate Predictor of Plasma Lipid Phenotypes Following Roux‐en‐Y Gastric Bypass in Women: An Exploratory Analysis of the SURMetaGIT Study

**DOI:** 10.1155/jobe/2335829

**Published:** 2026-08-12

**Authors:** Gabriela de O. Lemos, Raquel S. M. Torrinhas, Jéssica M. Volejnik Pino, Philip C. Calder, Steven B. Heymsfield, Natasha M. Machado, Dan L. Waitzberg

**Affiliations:** ^1^ Departamento de Gastroenterologia, Universidade de São Paulo, São Paulo, São Paulo, Brazil, usp.br; ^2^ Escola Paulista de Medicina, Universidade Federal de São Paulo, São Paulo, São Paulo, Brazil, unifesp.br; ^3^ School of Human Development and Health, University of Southampton, Southampton, UK, southampton.ac.uk; ^4^ NIHR Southampton Biomedical Research Centre, Southampton, UK; ^5^ Pennington Biomedical Research Foundation, Baton Rouge, Louisiana, USA

**Keywords:** bariatric surgery, cholesterol, metabolomics, Roux-en-Y gastric bypass, sphingolipids

## Abstract

Roux‐en‐Y gastric bypass (RYGB) surgery is known to improve lipid profiles in individuals with obesity, but the degree of improvement varies. Emerging evidence suggests a role for sphingolipids in modulating cholesterol metabolism. This study investigated the relationship between plasma sphingolipid changes and cholesterol improvements in women with obesity and Type 2 diabetes mellitus (T2DM) following RYGB. Patients from the SURMetaGIT study were evaluated before and 3 months after surgery. Biochemical and sphingolipid analyses, anthropometric measures, and body composition were assessed. A nonhierarchical cluster analysis assessed distinct phenotypes in response to cholesterol after surgery. The *p* value from multiple comparison tests was adjusted using the false discovery rate (FDR) method. A metabolite fold change [log_2_ (postoperative mean/preoperative mean)] of 1.2 and an adjusted *p* value < 5% were considered statistically significant. A linear regression model was used to investigate the relationship between sphingolipids and cholesterol levels. Finally, a binary logistic regression model was developed to identify the predictive value of specific discriminative sphingolipids in the lipid improvement after RYGB. Distinct sphingolipid remodeling patterns were observed, with specific sphingolipids, including Cer(d18:1/24:0), SM(d18:1/22:0), and SM(d18:1/24:0), presenting a strong positive correlation with cholesterol levels, independent of weight loss and T2DM remission. The binary logistic regression showed preoperative Cer(d18:1/24:0) could be the strongest candidate predictor of plasma lipid improvement after surgery (accuracy of 78%; AUC 0.87). These findings suggest that specific sphingolipids, notably preoperative Cer(d18:1/24:0), seemed to have a potential role in predicting cholesterol improvement after RYGB. External validation in independent cohorts is warranted before clinical applicability can be established.

**Trial Registration:** ClinicalTrials.gov identifier: NCT01251016

## 1. Introduction

Metabolic and bariatric surgery (MBS) is widely recognized as the most effective intervention for weight loss and glycemic control in individuals with obesity and Type 2 diabetes mellitus (T2DM) [1]. Evidence from robust systematic reviews and meta‐analyses highlights the extensive benefits of MBS, which go beyond weight reduction and T2DM management. These benefits include improvements in osteoarthritis and nonalcoholic fatty liver disease and reductions in overall cancer risk and mortality [2–5]. Additionally, MBS contributes to better end‐organ microvascular health and the regression of diabetes‐related complications, resulting in a decline in cardiovascular mortality [6, 7].

Despite the significant cardiovascular benefits, the effects of different MBS procedures on blood lipid profiles vary, and the reasons for these differences remain unclear [8]. Addressing this knowledge gap is critical, given the strong association between serum cholesterol—particularly the pool of proatherogenic fractions, compiled as non–high‐density lipoprotein cholesterol (non–HDL‐c)—and atherosclerosis, a major contributor to cardiovascular risk [9, 10]. The causal link between cholesterol and cardiovascular disease (CVD) is further supported by the success of lipid‐lowering therapies in reducing cardiovascular events [11].

Beyond cholesterol, certain sphingolipids have emerged as important factors influencing cardiovascular risk in individuals with T2DM and in the general population, particularly saturated long‐chain and very long‐chain ceramides, such as Cer(d18:1/16:0), Cer(d18:1/22:0), Cer(d18:1/24:0), and Cer(d18:1/24:1) [12–15]. Sphingolipids are a diverse group of lipids, primarily composed of ceramides (Cer), which consist of a sphingoid base linked to a fatty acid via an amide bond. Sphingolipid structures are further defined by the head group attached to the ceramide backbone, such as the phosphorylcholine head group in sphingomyelins (SMs) or the sugar head group in glycosphingolipids [16].

In eukaryotic cells, sphingolipids and cholesterol are key components of lipid rafts—specialized microdomains involved in cell signaling processes related to diseases such as obesity, insulin resistance, nonalcoholic fatty liver disease, and T2DM [16–18]. The SM‐to‐cholesterol ratio in the plasma membrane influences cholesterol metabolism, potentially affecting its circulating levels [19]. Alterations in SM synthesis or sphingomyelinase (SMase) activity in the plasma membrane can decrease the activity of 3‐hydroxy‐3‐methylglutaryl‐coenzyme A (HMG‐CoA) reductase, resulting in decreased plasma cholesterol and triglycerides and altered lipoprotein distribution [19–21]. Inhibition of ceramide synthesis also downregulates sterol‐regulatory element‐binding proteins (SREBPs), further decreasing HMG‐CoA reductase activity, independent of SM [22, 23].

Following Roux‐en‐Y gastric bypass (RYGB), changes in blood lipid profiles are often observed independent of weight loss [24]. Cholesterol and sphingolipid remodeling appear to differ based on the type of MBS performed, specifically whether it is restrictive or mixed. While the mechanisms underlying these changes are not fully understood, many studies report an increase in unsaturated SMs and a decrease in saturated SMs post‐MBS, although findings are inconsistent [25]. Our group has demonstrated that certain saturated long‐chain SMs exhibit a positive correlation with cholesterol and its fractions following MBS [26]. However, the specific role of plasma sphingolipid types in shaping the plasma lipid response to MBS still needs to be better understood. In this exploratory study, we hypothesized that enhanced cholesterol metabolism after RYGB would be associated with changes in specific sphingolipid species. Therefore, we aimed to analyze the relationship between the circulating cholesterol profile and changes in specific sphingolipid species in a cohort of women undergoing RYGB.

## 2. Materials and Methods

### 2.1. Study Design and Participants

This is an exploratory post hoc analysis of the SURMetaGIT study. All research procedures, including sample size calculations, are fully described elsewhere, and the trial is registered at https://www.clinicaltrials.gov [27]. The protocol was based on the STROBE statement for cohort studies and was approved by the local ethics committee (CAPPesq, n° 6.054.077). Thirty patients were enrolled between April 2012 and March 2016. Given the small sample size and the disproportionately higher female sex enrolled for bariatric surgery at the department (∼70%, which aligns with epidemiologic studies [28, 29]), only adult biological women (ages between 18 and 60 years) undergoing RYGB at the Bariatric and Metabolic Surgery Department at the Hospital das Clínicas of the University of São Paulo Medical School were included in the SURMetaGIT study to decrease sample heterogeneity. All participants underwent RYGB with standard techniques (gastric pouch ∼30 mL, biliopancreatic limb ∼50–60 cm, and alimentary limb ∼100–120 cm).

Inclusion criteria were as follows: Grade II or III obesity (35 ≤ body mass index [BMI] ≤ 50 kg/m^2^), T2DM diagnosis (fasting plasma glucose ≥ 126 mg/dL or HbA1c ≥ 7% or antidiabetic drug use) before surgery, and agreement to participate with free and informed consent. Exclusion criteria included liver dysfunction, thyroid dysfunction, abdominal hernia, *Helicobacter pylori* infection, insulin use, and enrollment in other studies. Individuals were evaluated at the baseline and 3 months after surgery for blood collection, body composition, and anthropometric assessment.

### 2.2. Body Composition Assessment

Body composition was analyzed using air displacement plethysmography (BOD POD, COSMED, USA) and is expressed in kilograms or as a percentage. Body weight (kilograms) and height (centimeters) were measured using an electronic scale (Lucastec, Brazil) and a stadiometer (Sanny, American Medical of Brazil), respectively. Waist and hip circumferences were measured using an inelastic tape and are expressed in centimeters. BMI and body roundness index (BRI), a shape measure associated with the metabolic phenotype [30], were calculated as body weight (in kilograms) divided by height squared (in meters) and 364.2 − 365.5 ∗ √[1 − (WC/(2*π*))^2/(0.5 ∗ height)^2], respectively [30].

### 2.3. Plasma Collection and Analysis

Blood samples were collected into EDTA‐containing tubes following a 12‐h fasting period and immediately sent to the Central Laboratory of HC‐FMUS, where biochemical analyses were performed at baseline and 3 months after surgery. Information regarding equipment, companies, reagents, and laboratory references can be obtained at https://patologiaclinica.hc.fm.usp.br/lista-manual-exames. Briefly, fasting plasma glucose was measured by the hexokinase enzymatic method, HbA1c was measured by ion exchange high‐performance liquid chromatography–Variant II, and insulin and C‐peptide levels were assessed by electrochemiluminescence immunoassay. Total cholesterol (TC) and its fractions were measured using the colorimetric enzymatic cholesterol oxidase/peroxidase–aminoantipyrine method. Non–HDL‐c was calculated using the following formula: non–HDL‐c = total cholesterol (TC) – high‐density lipoprotein (HDL‐c). The remission of Type 2 diabetes was determined based on fasting glucose and HbA1c levels, according to the 2021 criteria of the American Diabetes Association (ADA) [31].

### 2.4. Sphingolipid Profiling

Blood samples (*n* = 28 participants) were centrifuged at 2800 rpm and 4°C for 10 min, and the supernatant plasma was transferred to microtubes and stored at −80°C until analysis. Plasma sphingolipids were extracted using a methanol‐based aqueous solution and analyzed by untargeted metabolomics using ultraperformance liquid chromatography coupled to mass spectrometry (UPLC–MS; Agilent 6530 Accurate‐Mass Q‐TOF LC/MS and Agilent 1290 Infinity II LC System with Charged Surface Hybrid [CSH] column, Agilent Technologies MS) at the National Institute of Health West Coast Metabolomics Center, Davis, CA, USA. Samples were analyzed in a randomized fashion, with quality control samples inserted every 10 samples. Quality controls included samples with known metabolite concentrations (Bioreclamation IVT; Westbury, NY, USA) to monitor instrument stability, analytical reproducibility, signal drift, and overall data quality throughout the acquisition phase. Data were processed and analyzed using MS‐DIAL software (https://prime.psc.riken.jp/Metabolomics_Software/). Sphingolipids were annotated by matching accurate mass, retention time, and MS/MS spectra against in‐house libraries, the Fiehn laboratory databases, and authenticated reference standards at the West Coast Metabolomics Center. Metabolites presenting a coefficient of variation (CV) > 20% or detected in fewer than 50% of samples were excluded. Sphingolipids with the highest CV were excluded when identified in both positive and negative ionization modes.

### 2.5. Statistical Analysis

The present study retrospectively analyzed data on the plasma abundance of sphingolipids identified in the SURMetaGIT study. The original sample size (*n* = 20) of this cohort was estimated based on transcriptomic outcomes, with 10 additional participants further included for gene expression validation. The full description of sample size estimation is described elsewhere [27]. Considering 32 identified sphingolipids and a paired study design (pre‐ and postoperative analysis), a nonparametric paired test adjusted for multiple hypothesis tests using the false discovery rate (FDR) was performed to detect metabolites with statistical significance, considering an adjusted *p* value of 0.05. The statistical power was calculated using the SSPA package available in MetaboAnalyst 6.0, considering multiple comparison tests and an FDR of 0.03.

Data regarding age, lipid‐lowering therapy use, and history of previous cholecystectomy (absence of the gallbladder) were collected to assess confounding variables that could influence the changes in cholesterol and sphingolipids.

A nonhierarchical cluster analysis was performed in R Studio using the K‐means algorithm (postsurgery very‐low‐density lipoprotein [VLDL‐c]/presurgery VLDL‐c) to test whether patients exhibit distinct circulating cholesterol responses to RYGB. VLDL‐c was chosen for cluster segregation due to the most pronounced role of sphingolipids on the synthesis of this cholesterol subfraction, as demonstrated by Tae‐Sik Park et al. [20], for its potential to exhibit clinically significant proatherogenic activity [32] and for its ability to segregate the groups properly, as demonstrated by the silhouette width of 0.58 in the clustering structure, suggesting satisfactory separation between the formed groups and adequate internal cohesion of clusters [33] (Supporting information A). Changes in body composition and biochemical variables were calculated as delta values ([post – pre]/pre) and are reported as ratios or relative values.

Exploratory analysis of sphingolipids in each cluster was performed using MetaboAnalyst Versions 5.0 and 6.0 and included volcano plots and heatmaps. For the volcano plots, data were autoscaled, and *p* values were adjusted using the FDR method to control for multiple comparisons across sphingolipids identified (Supporting Information B). The fold change cutoff was ±1.2, defined considering the paired study design and limited sample size [34]. Fold change was calculated as log_2_ (postoperative mean/preoperative mean). A 5% significance level was adopted for adjusted *p* values.

Inferential statistical analysis was performed using Jamovi software, Version 2.2.5. Continuous variables are reported as medians and interquartile ranges, and categorical data are presented as absolute and relative frequencies, given the non‐normal distributions indicated by the Shapiro–Wilk test. When variables were normally distributed, they were marked with an asterisk and reported as the mean and standard deviation. For categorical data, intergroup differences were assessed before and after surgery using Fisher’s exact test. For continuous variables, intragroup comparisons were assessed by the Wilcoxon test and intergroup changes by Student’s *t*‐test or the Mann–Whitney test, according to data distribution. The effect size (ES) was assessed using Cohen’s d test or the rank‐biserial correlation for normally and non‐normally distributed variables, respectively. Spearman’s correlation was used for correlation analysis. For discriminative sphingolipids that showed a strong correlation with cholesterol levels, a simple linear regression analysis was performed to assess their influence on TC and its subfractions. Residuals’ linearity, homoscedasticity, and normality assumptions were verified. This representation was preferred given the sample size, and a generalized linear model (GLM) analysis was employed whenever the linear regression assumptions were not met.

A binary logistic regression was used to estimate the association between the preoperative levels of discriminative sphingolipids with stronger predictive capacity, determined by the linear analysis, and the improvement in the lipid profile after RYGB, considered as non–HDL‐c < 100 mg/dL [32]. In addition, T2DM remission, lipid‐lowering therapy, and previous cholecystectomy were independently adjusted within the model to control for confounding variables (Supporting Information C). ROC curves, along with calculations of accuracy, sensitivity, specificity, and the area under the curve (AUC), were used to evaluate the discriminative capacity of the models. Β coefficient, *p* value, McFadden’s *R* ^2^, and Akaike information criterion (AIC) were also reported. For all statistical tests, a significance level of *p* < 0.05 was adopted. Analyses were conducted using Jamovi software.

## 3. Results

**FIGURE 1:**
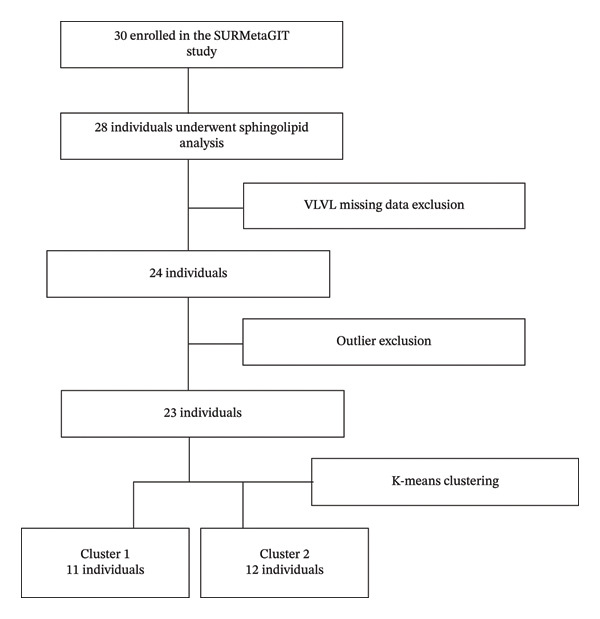
Flow diagram of patient inclusion. VLDL: very‐low‐density lipoprotein. K‐means cluster analysis was performed in R Studio using the algorithm (postsurgery VLDL‐c/presurgery VLDL‐c), with a silhouette width of 0.58 in clustering structure.

Of the 30 patients enrolled in the SURMetaGIT study, 28 underwent untargeted plasma metabolomic analysis, and their sphingolipid profiles were assessed. Four patients were excluded because VLDL‐c analysis was missing either before or within 3 months after surgery, and one patient was excluded as an outlier due to abnormal and unexpected lipid changes after surgery. Cluster analysis identified two lipid phenotypes, dividing the population into Cluster 1 (*n* = 11) and Cluster 2 (*n* = 12). The effective sample size (*n* = 23) yielded an estimated statistical power of 80%, considering multiple comparison tests and an FDR of 0.03. The patient workflow is shown in Figure 1.

The baseline characteristics of the two clusters are presented in Table 1. Before surgery, both groups had similar age (47 years [Cluster 1] vs. 45 years [Cluster 2], *p* = 0.35) and anthropometric parameters. Cluster 2 exhibited higher levels of VLDL‐c and triglycerides prior to surgery.

**TABLE 1:** Baseline characteristics of the two phenotypic clusters before surgery.

	Cluster 1		Cluster 2		*p* value	Effect size
	Median	IQR	Median	IQR		
Age (years)	47	6.9	45	6.3	0.35	—
Weight (kg)^∗^	112.3	19.9	115.7	13.2	0.63	0.20
BMI (kg/m^2^)^∗^	44.3	6.5	47.1	4.9	0.24	0.49
Arm circumference (cm)^∗^	42.8	5.5	42.2	4.9	0.76	0.12
Waist circumference (cm)^∗^	121.4	12.6	130.1	10.6	0.08	0.76
Hip circumference (cm)^∗^	137.4	14.0	139.6	10.8	0.68	0.17
Waist–hip ratio^∗^	0.89	0.08	0.93	0.08	0.17	0.58
Fat mass (kg)^∗^	57.7	17.8	61.5	10.4	0.54	0.26
Lean mass (kg)^∗^	53.6	8.2	53.4	7.9	0.95	0.02
BRI	10.3	2.7	11.6	2.9	0.46	0.06
Glucose (mg/dL)	179.0	118.0	206.0	118.0	0.525	0.17
Insulin (μU/mL)	17.30	12.50	17.95	15.9	0.97	0.02
HbA1c (%)	8.00	2.90	9.15	2.0	0.22	0.32
C‐peptide (ng/mL)	4.35	1.20	3.65	2.6	0.55	0.16
Total cholesterol (mg/dL)	198.0	64.0	192.0	20.25	0.71	0.1
HDL‐c (mg/dL)	48.0	18.0	43.5	10.25	0.17	0.34
Non–HDL‐c (mg/dL)	139.0	54.0	148.5	21.25	1.00	0.00
LDL‐c (mg/dL)	117.0	42.0	114.0	22.75	0.28	0.27
VLDL‐c (mg/dL)	21.0	10.0	30.0	15.0	**0.01**	**0.64**
Triglycerides (mg/dL)	104.0	49.0	150.0	75.25	**0.01**	**0.64**

*Note:* HbA1c: glycated hemoglobin; HDL‐c: high‐density lipoprotein; LDL‐c: low‐density lipoprotein. Differences with statistical significance are expressed in bold. The asterisk (^∗^) demarcates data with a normal distribution, thus presented as mean and standard deviation, instead of median and interquartile range.

Abbreviations: BMI: body mass index; BRI: body roundness index; VLDL: very‐low‐density lipoprotein.

Because both cholecystectomy and the use of lipid‐lowering medications influence plasma cholesterol levels [35], and cholecystectomy has previously been associated with alterations in plasma metabolite profiles in this cohort [36], we compared these 2 variables in the two clusters. The absence of the gallbladder (27% [Cluster 1] vs. 25% [Cluster 2], *p* = 0.9) and the use of lipid‐lowering therapy (64% [Cluster 1] vs. 25% [Cluster 2], *p* = 0.1) did not differ between clusters before surgery.

Three months after surgery, Cluster 2 showed an increased incidence of T2DM remission compared with Cluster 1, although this difference did not reach statistical significance (83% vs. 64%, *p* = 0.3). The changes in body weight and composition after surgery were similar between clusters. All biochemical parameters exhibited similar changes between clusters, except for TC, non–HDL‐c, VLDL‐c, and triglycerides, where the effects were more pronounced in Cluster 2. Differences among these parameters between clusters after surgery (delta values) are shown in Table 2 and expressed as ratios or relative values.

**TABLE 2:** Comparison of changes in body weight and composition and biochemical parameters between clusters 3 months after surgery.

	Cluster 1		Cluster 2		*p* value	Effect size
	Median	IQR	Median	IQR		
Weight (kg)	−0.18	0.01	−0.19	0.05	0.28	0.27
BMI (kg/m^2^)	−0.18	0.01	−0.19	0.05	0.30	0.25
Arm circumference (cm)	−0.13	0.09	−0.10	0.15	0.78	0.07
Waist circumference (cm)	−0.10	0.07	−0.12	0.03	0.37	0.22
Hip circumference (cm)	−0.11	0.05	−0.11	0.04	0.71	0.09
Waist–hip ratio	0.01	0.03	−0.01	0.07	0.87	0.04
Fat mass (kg)	−0.29	0.12	−0.31	0.04	0.33	0.25
Lean mass (kg)	−0.04	0.04	−0.06	0.10	0.74	0.09
BRI	−0.22	0.13	−0.24	0.06	0.28	0.27
Glucose (mg/dL)	−0.46	0.23	−0.51	0.11	0.34	0.24
Insulin (μU/mL)	−0.63	0.45	−0.43	0.24	0.42	0.22
HbA1c (%)	−0.25	0.21	−0.36	0.06	0.17	0.36
C‐peptide (ng/mL)	−0.40	0.08	−0.16	0.25	0.44	0.19
Total cholesterol (mg/dL)	−0.17	0.35	−0.27	0.10	**0.01**	**0.59**
HDL‐c (mg/dL)	−0.11	0.16	−0.10	0.26	0.74	0.09
Non–HDL‐c (mg/dL)	−0.13	0.51	−0.36	0.12	**0.01**	**0.62**
LDL‐c (mg/dL)	−0.17	0.54	−0.29	0.18	0.26	0.28
VLDL‐c (mg/dL)	0.05	0.01	−0.43	0.29	**< 0.001**	**1.00**
Triglycerides (mg/dL)	0.05	0.17	−0.44	0.28	**< 0.001**	**1.00**

*Note:* HbA1c: glycated hemoglobin; HDL‐c: high‐density lipoprotein; LDL‐c: low‐density lipoprotein. Delta values ([post − pre]/pre) are expressed as ratios or relative values. Statistically significant differences are expressed in bold.

Abbreviations: BMI, body mass index; BRI, body roundness index; VLDL, very‐low‐density lipoprotein.

**FIGURE 2:**
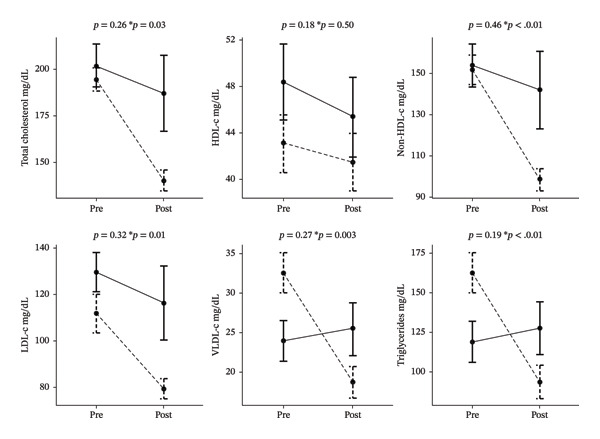
Lipid profile before and 3 months after RYGB. HDL‐c: high‐density lipoprotein; LDL‐c: low‐density lipoprotein; VLDL: very‐low‐density lipoprotein. Preoperative and postoperative lipid levels are shown, along with their confidence intervals. Lipid changes in Cluster 1 are shown in the full line; lipid changes in Cluster 2 are shown in the dashed line. *p* values indicate the significance of the post‐ vs. presurgery comparison within each cluster. The *p* value of Cluster 2 is marked with asterisks. For intergroup delta values comparisons, refer to Table 2.

The modification in the plasma lipid profile 3 months after surgery (TC, non–HDL‐c, low‐density lipoprotein [LDL‐c], VLDL‐c, and TGs) is shown in Figure 2. Although both groups experienced similar changes in body weight and composition, only Cluster 2 showed an improvement in plasma lipid levels. This finding is further supported by the greater delta variation observed in Cluster 2 for lipid parameters, as demonstrated in Table 2.

**FIGURE 3:**
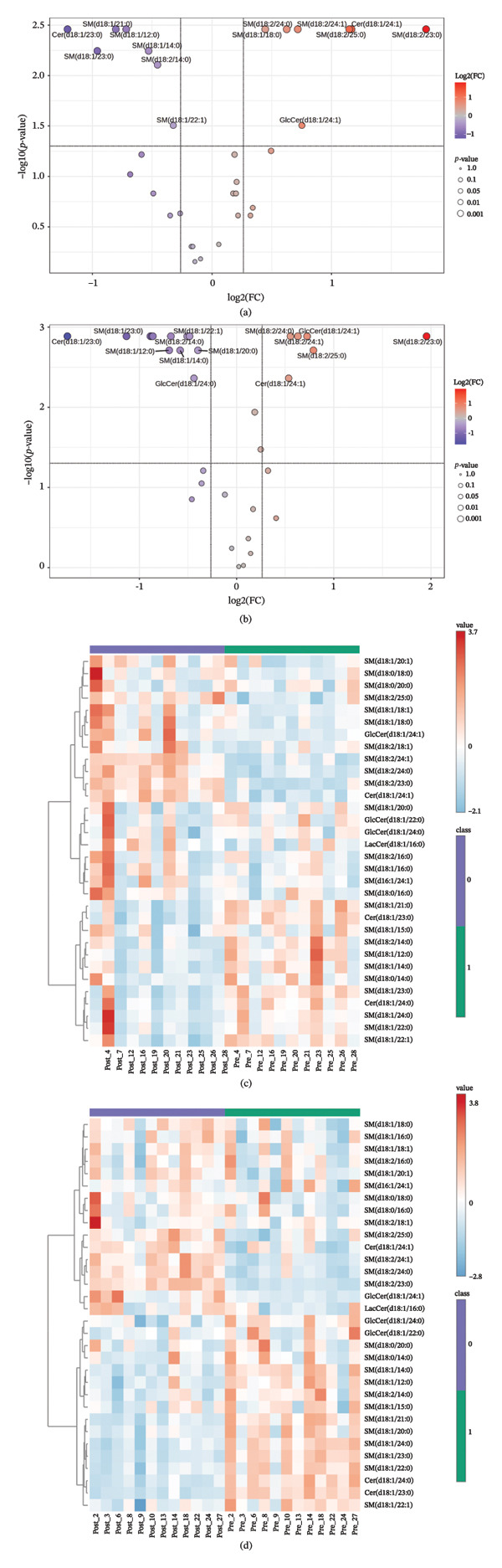
Sphingolipids’ abundance before and after RYGB. Volcano plot of significantly increased (in red) or decreased (in blue) sphingolipids (adjusted *p* value < 0.05) identified in Clusters 1 (a) and 2 (b); heatmap showing relative abundance across participants before (right side, under the green box) and 3 months after (left side, under the blue box) surgery in Clusters 1 (c) and 2 (d).

After data processing and curation, 32 sphingolipids were identified in plasma samples, comprising 3 Cer, 4 glycosphingolipids, and 25 SMs (Supporting Information B). Sphingolipids’ abundance identified in Clusters 1 and 2 is shown in the volcano plots and heatmaps in Figure 3.

**FIGURE 4:**
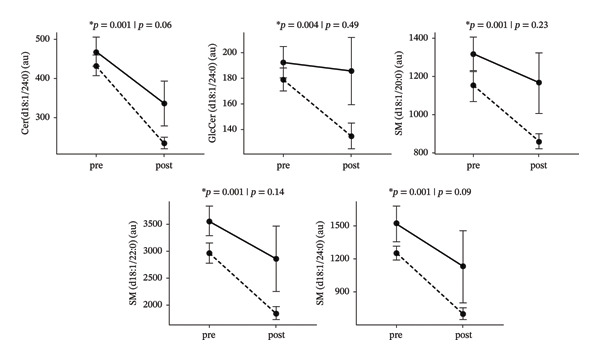
Changes of discriminative sphingolipids after RYGB. Cer: ceramide; GlcCer: glucosyl ceramide; SM: sphingomyelin. Preoperative and postoperative sphingolipid abundance is shown, along with their confidence intervals. Sphingolipid changes in Cluster 1 are shown in the full line; sphingolipid changes in Cluster 2 are shown in the dashed line. *p* values indicate the significance of the post‐ vs. presurgery comparison within each cluster. The *p* value of Cluster 2 is marked with asterisks.

Clusters 1 and 2 presented 14 and 18 sphingolipids that changed significantly after surgery, respectively. Among these, 5 differentially changed in Cluster 2 (encompassing patients who presented an improvement in the lipid profile) with medium to large ES: Cer(d18:1/24:0) ES 1.0; glucosyl ceramide (GlcCer) GlcCer(d18:1/24:0) ES 0.8; SM(d18:1/20:0) ES 0.9; SM(d18:1/22:0) ES 1.0; and SM(d18:1/24:0) ES 1.0 (Figure 4).

Considering the sphingolipids that changed only in the group with lipid improvement (Cluster 2), hereafter referred to as discriminative sphingolipids, we evaluated whether these metabolites were associated with plasma lipid parameters. In this regard, Table 3 shows the correlation between the discriminative sphingolipids and plasma cholesterol and its subfractions in both the pre‐ and postoperative periods. Moderate to strong correlations were found between TC, non–HDL‐c, LDL‐c, and HDL‐c and the following five sphingolipids: Cer(d18:1/24:0), GlcCer(d18:1/24:0), SM(d18:1/20:0), SM(d18:1/22:0), and SM(d18:1/24:0), particularly after surgery (Table 3).

**TABLE 3:** Correlation between discriminative sphingolipids from Cluster 2 and lipid profile.



*Note:* a) Correlation between discriminative sphingolipids from Cluster 2 and lipid profile, both measured before surgery; b) Correlation between discriminative sphingolipids from Cluster 2 and lipid profile, both measured after surgery. HDL‐c: high‐density lipoprotein; LDL‐c: low‐density lipoprotein. Values are Spearman’s correlation coefficients. Statistically significant differences are expressed in bold. Negative correlations are presented in red color, and positive correlations are expressed in blue color. The more intense colors in the boxes represent stronger correlations.

Abbreviation: VLDL, very‐low‐density lipoprotein.

^∗^ *p* < 0.05.

^∗∗^ *p* < 0.01.

^∗∗∗^ *p* < 0.001.

In light of the significant correlations found, a simple linear regression analysis was performed to determine whether these discriminative sphingolipids could predict changes in plasma cholesterol and its fractions, thereby identifying which metabolites exert a greater influence on the lipid response to RYGB. According to simple linear regression, discriminative sphingolipids showed a significant impact on TC and its subfractions, especially Cer(d18:1/24:0), SM(d18:1/22:0), and SM(d18:1/24:0). These three sphingolipids were identified to be the candidate predictors of higher TC, non–HDL‐c, and LDL‐c levels before surgery: SM(d18:1/24:0) accounted for 48% of the change in TC, 38% in non–HDL‐c, and 23% in LDL‐c; Cer(d18:1/24:0) accounted for 45% of the change in TC, 47% in non–HDL‐c, and 24% in LDL‐c; SM(d18:1/22:0) accounted for 42% of the change in TC, 30% in non–HDL‐c, and 20% in LDL‐c. A less pronounced effect was seen for SM(d18:1/20:0), which accounted for 26% of the change in TC and 20% in HDL‐c; and GlcCer(d18:1/24:0), which accounted for 15% of the change in TC and 10% in non–HDL‐c (Supporting Information D).

The predictive performance of Cer(d18:1/24:0), SM(d18:1/22:0), and SM(d18:1/24:0), assessed through preoperative ROC curve analysis, showed an apparent AUC of 0.87 for Cer(d18:1/24:0) and 0.78 for both SM(d18:1/22:0) and SM(d18:1/24:0), suggesting preliminary discriminative potential between patients who improved their lipid profile after surgery and those who did not. Cer(d18:1/24:0) showed an apparent accuracy of 78%, with a sensitivity of 77% and specificity of 80%, whereas SM(d18:1/22:0) and SM(d18:1/24:0) showed an apparent accuracy of 70%, sensitivity of 77%, and specificity of 60.0% (Supporting Information E). Given the limited sample size and the absence of external validation, these findings should be interpreted cautiously and considered exploratory, requiring confirmation in larger independent cohorts.

The binary logistic regression analysis suggested that higher preoperative plasma levels of SM(d18:1/22:0) were associated with slightly lower odds of not achieving a favorable lipid response after surgery (OR = 0.998; 95% CI: 0.99–0.99; *p* = 0.048). In contrast, SM(d18:1/24:0) showed a borderline association with higher odds of nonresponse (OR = 1.006; 95% CI: 0.99–1.01; *p* = 0.050). On the other hand, Cer(d18:1/24:0) showed the most consistent association with the absence of a favorable lipid response (OR = 1.019; 95% CI: 1.00–1.03; *p* = 0.028). It also presented slightly better predictive performance than the other lipids analyzed, based on AUC, specificity, accuracy, and McFadden *R* ^2^ values (Table 4). However, these findings should be interpreted with caution due to the exploratory nature of the study and the small sample size.

**TABLE 4:** Binary logistic regression models evaluating preoperative sphingolipids and lipid response 3 months after surgery.

	SM(d18:1/22:0)	SM(d18:1/24:0)	Cer(d18:1/24:0)
*β* (estimate)	0.0018	0.0063	0.0195
Odd ratio	0.998	1.006	1.019
95% CI	0.99–0.99	0.99–1.01	1.00–1.03
*p*‐value	0.048	0.050	0.028
McFadden *R* ^2^	0.205	0.274	0.315
AIC	29.02	26.87	25.56
Accuracy	70%	70%	78%
Sensitivity	77%	77%	77%
Specificity	60%	60%	80%
AUC	0.7846	0.777	0.869

*Note:* Cer: ceramide; SM: sphingomyelin. 95% CI: 95% confidence interval.

Abbreviations: AIC, Akaike information criterion; AUC, area under the curve.

After adjusting these variables for potential covariates in the binomial logistic regression models (T2DM, medication use, and cholecystectomy), only cholecystectomy showed a significant association with higher odds of a worsening lipid profile in the models containing SM(d18:1/22:0) (OR = 97.326; *p* = 0.029) and SM(d18:1/24:0) (OR = 31.786; *p* = 0.034), while it showed a borderline association in the model with baseline ceramide (OR = 35.204; *p* = 0.076). However, the wide confidence intervals observed for this covariate suggest that these findings should be interpreted with caution (Supporting Information C). Nevertheless, none of the potential covariates substantially change the direction or magnitude of the observed associations. In addition, these variables showed no significant differences between groups in our samples.

## 4. Discussion

The findings of this study align with previous reports of varied plasma lipid responses following MBS, while providing new insights into the underlying mechanisms regarding cholesterol metabolism. We observed differential improvements in circulating cholesterol and its fractions after RYGB that were not influenced by factors such as weight loss, body composition, lipid‐lowering therapy, or changes in glycemic control and T2DM remission. Instead, these improvements were associated with specific sphingolipid responses, notably the long‐chain sphingolipids Cer(d18:1/24:0), SM(d18:1/22:0), and SM(d18:1/24:0).

The reduction in non–HDL‐c, which encompasses the most atherogenic cholesterol fractions, including VLDL‐c and LDL‐c, emerged as a key factor in improved cholesterol homeostasis following MBS. Given that T2DM is associated with an elevated risk of CVD [37], non–HDL‐c serves as a primary risk marker in both T2DM patients and in the general population [9]. Non–HDL‐c levels are recommended to be below 130 mg/dL for moderate‐risk individuals, 100 mg/dL for high‐risk individuals, and 85 mg/dL for very high‐risk individuals [37]. In our cohort of women with obesity and T2DM, baseline non–HDL‐c levels exceeded the recommended thresholds in both clusters. However, women in Cluster 2 achieved significantly lower non–HDL‐c levels 3 months postsurgery, reaching a median value of under 100 mg/dL, indicating a differential benefit for this high‐risk population.

Despite the well‐documented heterogeneity in lipid homeostasis changes after MBS, our study highlights that these alterations occur independently of weight loss. The observed median changes in cholesterol fractions varied between clusters, with Cluster 2 presenting more pronounced improvements. Although the expected benefits in HDL‐c levels were not observed in either cluster (+2 mg/dL and −4 mg/dL for Clusters 1 and 2, respectively), the current study did not evaluate the HDL‐c particle size, thus not addressing changes in HDL‐c quality. Moreover, the significant reduction in the pool of proatherogenic cholesterol fractions in Cluster 2 aligns with better cardiovascular outcomes in this group, suggesting that only a subset of patients may experience this beneficial effect of MBS.

There is limited research on the role of sphingolipid remodeling following MBS. Cer have been described as primary lipotoxic players in metabolic diseases [38]. Long‐chain Cer, such as Cer(d18:1/24:0), have been incorporated into cardiovascular risk screening scores to enhance the prediction of cardiovascular complications [39, 40]. A meta‐analysis of seven cohort studies, encompassing nearly 30,000 individuals, identified high‐risk Cer for major cardiovascular events, such as Cer(d18:1/16:0), Cer(d18:1/18:0), and Cer(d18:1/24:1) [13]. In our study, only one high‐risk ceramide [Cer(d18:1/24:1)] was identified. This molecule increased postsurgery in both clusters but with a more prominent increase in Cluster 1 [1.17 vs. 0.54 log2(FC)]. Cer(d18:1/24:0), which has no definite association with major cardiovascular events, and decreased significantly only in Cluster 2 [13].

Given their structural integration in lipid rafts, the SM‐to‐cholesterol ratio may significantly influence cholesterol metabolism and the regulation of plasma lipoproteins. Inhibition of SM synthesis has been linked to reductions in plasma cholesterol and triglycerides [20]. Conversely, increased SM synthesis is associated with VLDL‐c production in hepatocytes and atherogenic processes, as well as being linked to reverse cholesterol transport [41]. Although these findings appear contradictory, there might be a role for specific SM species in the metabolism of cholesterol‐rich lipoproteins that remains unclear.

Our study identified specific SM species, such as SM(d18:1/22:0) and SM(d18:1/24:0), that appear to have a promising apparent discriminative ability to predict circulating cholesterol changes. The significant reduction of SM(d18:1/22:0) was linked to improvements in the plasma cholesterol profile, whereas other similar SMs with the same number of carbons (40), but different structures, such as SM(d18:1/22:1) and SM(d16:1/24:1), did not show the same association. The distinct structural differences between these SMs may explain their varying roles in cholesterol metabolism, given their involvement in cell signaling through the lipid rafts [42]. Notably, preoperative SM(d18:1/24:0) levels accounted for nearly 50% of the variance in TC before surgery. Several mechanisms can influence sphingolipid synthesis through its rate‐limiting enzyme, serine palmitoyl‐CoA transferase (SPT). Factors such as the availability of fatty‐acyl CoA, lipopolysaccharide (LPS) activity, toll‐like receptor 4 (TLR4) activation, tumor necrosis factor‐α (TNF‐α), cortisol levels, and the composition of the gut microbiota play a role in this process [41, 43, 44]. Notably, gut bacteria from the *Bacteroides* genus express SPT, and sphingolipids produced by these bacteria can be absorbed and metabolized by the host [45]. Previous research from our group has shown that mechanisms such as reduced fatty‐acyl CoA substrates, decreased TLR4 activation, and changes in the gut microbiota (e.g., a lower Firmicutes‐to‐Bacteroidetes ratio) are seen in this cohort post‐RYGB; therefore, these mechanisms may contribute to the observed alterations in sphingolipids [36, 46, 47].

Additionally, GlcCer(d18:1/24:0), a cerebroside known as ganglioside GL1a, was identified as positively correlating with cholesterol metabolism [48]. Cholesterol‐rich lipoproteins like VLDL and LDL are enriched with this sphingolipid, and their levels are in equilibrium with red blood cells, which potentially decrease after RYGB due to enhanced red blood cell turnover [36]. This turnover may contribute to changes in cholesterol metabolism, as observed in conditions like Gaucher Disease, where decreased levels of cholesterol fractions are reported [48]. However, the exact role of red blood cell turnover and ganglioside GL1a in cholesterol metabolism requires further investigation.

Our preliminary findings should be interpreted with caution due to several limitations that may impact the generalizability of the results. First, the post hoc, exploratory nature of this study; second, the inclusion of female patients only; third, the small sample size, not primarily designed for the outcomes reported here; fourth, the lack of a control group that may limit our ability to definitively attribute observed changes in lipid profiles to surgical intervention specifically; fifth, the short follow‐up period of 3 months postsurgery likely does not capture the full extent of lipid metabolism changes following RYGB, underscoring the need for longer‐term assessments. Additionally, excluding participants with missing data or certain comorbidities may introduce selection bias, potentially affecting the study’s conclusions. Also, the lack of internal or external validation may lead to an overestimation of the model’s true discriminative ability. In this sense, we recommend further studies in larger cohorts as a key next step to validate our findings.

Finally, while our lipidomic analysis was comprehensive in its focus on sphingolipids and cholesterol metabolism, it did not include other lipid classes or metabolic pathways that may influence postsurgical outcomes. Moreover, factors such as variations in nutrient intake and gut microbiota composition, which were not examined in this study, could play a significant role in modulating lipid metabolism after surgery. Future research should address these gaps by incorporating longer follow‐up periods, control groups, and broader lipidomic analyses. By overcoming these limitations, subsequent studies can build on our findings to provide a more comprehensive understanding of the metabolic changes induced by RYGB.

## 5. Conclusions

Improvements in cholesterol homeostasis following RYGB showed considerable individual variability. Our study suggests that specific alterations in sphingolipids, particularly Cer(d18:1/24:0), SM(d18:1/22:0), and SM(d18:1/24:0), may significantly contribute to these metabolic changes and decreased cardiovascular risk after RYGB. By examining the detailed lipid profiles, we uncovered hidden variations in lipid metabolism among individuals with similar clinical characteristics, emphasizing the potential role of specific sphingolipid types in shaping the plasma lipid response to RYGB.

In women with obesity and T2DM undergoing RYGB, these findings provide a better understanding of the mechanism driving postoperative improvements in the plasma cholesterol profile. The observed changes in specific sphingolipids suggest that they could serve as potential biomarkers for predicting individual lipid responses to RYGB. These hypothesis‐generating findings require validation in larger, independent, multicenter cohorts before their clinical utility can be established. A deeper understanding of these mechanisms could pave the way for developing new diagnostic tools or targeted therapeutic strategies that optimize metabolic health in patients undergoing bariatric surgery.

**Nomenclature**
ADA: American Diabetes Association
BMI: Body mass index
BRI: Body roundness index
Cer: Ceramide
CSH: Charged surface hybrid
CV: Coefficient of variation
ES: Effect size
FDR: False discovery rate
GlcCer: Glucosyl ceramide
GLM: Generalized linear model
HDL: High‐density lipoprotein
HMG‐CoA: 3‐Hydroxy‐3‐methylglutaryl‐coenzyme A
LDL: Low‐density lipoprotein
LPS: Lipopolysaccharide
MBS: Metabolic and bariatric surgery
RYGB: Roux‐en‐Y gastric bypass
SM: Sphingomyelin
SMase: Sphingomyelinase
SPT: Serine palmitoyl‐CoA transferase
SREBPs: Sterol‐regulatory element‐binding proteins
T2DM: Type 2 diabetes mellitus
TC: Total cholesterol
TLR4: Toll‐like receptor 4
TNF‐α: Tumor necrosis factor‐α
VLDL: Very‐low‐density lipoprotein

## Author Contributions

Gabriela de O. Lemos contributed to the study conceptualization and design, data analysis tools, and wrote the original draft; Raquel S. M. Torrinhas contributed to the study design and analysis, project supervision, and critically reviewed the paper; Jéssica M. Volejnik Pino contributed to the study statistical analysis and critically reviewed the paper; Philip C. Calder critically reviewed the paper; Steven B. Heymsfield critically reviewed the paper; Natasha M. Machado contributed to the study design and analysis tool, data collection and curation, project supervision, and critically reviewed the paper; Dan L. Waitzberg conceived and designed the analysis, funding acquisition, project administration, and supervision, and critically reviewed the paper.

## Funding

This study is linked to project no. 2011/09612‐3 and was funded by the Fundação de Amparo à Pesquisa do Estado de São Paulo (FAPESP).

## Ethics Statement

This study was conducted in accordance with the Declaration of Helsinki and was approved by the local ethics committee (Comissão de Ética para Análise de Projetos de Pesquisa ‐ CAPPesq). Approval number: 6.054.077; approval date: May 11, 2023.

## Consent

Informed consent was obtained from all participants involved in the study.

## Conflicts of Interest

The authors declare no conflicts of interest.

## Supporting Information

Additional supporting information can be found online in the Supporting Information section.

## Supporting information


**Supporting Information 1** Supporting information A: K‐means cluster metrics; Supporting information B: Sphingolipids with statistical significance identified in plasma samples; Supporting information C: Adjusted binomial logistic regression model for confounding variables; Supporting information D: Linear regression of discriminative sphingolipids; Supporting information E: Characteristic (ROC) curve of Cer(d18:1/24:0), SM(d18:1/22:0), and SM(d18:1/24:0) to predict postsurgical lipid profile improvement in the preoperative period.

**Supporting Information 2** STROBE‐checklist‐cohort.

## Data Availability

Data are available on request from the authors.
