# Supplementary material for: Preoperative Cer(d18:1/24:0) Shows Promise as a Candidate Predictor of Plasma Lipid Phenotypes Following Roux‐en‐Y Gastric Bypass in Women: An Exploratory Analysis of the SURMetaGIT Study
Source: J Obes. 2026 Aug 12;2026:2335829. doi: 10.1155/jobe/2335829 (PMC13465188; doi:10.1155/jobe/2335829)
Supplement: Supplementary file 1 [file JOBE-2026-2335829-s002.zip › Supp A.docx]

**Supplementary material A: K-means cluster metrics**

K-means cluster analysis was performed in R Studio using the algorithm (post-surgery VLDL-c/pre-surgery VLDL-c), with a silhouette width of 0.58 in clustering structure
