# Supplementary material for: Preoperative Cer(d18:1/24:0) Shows Promise as a Candidate Predictor of Plasma Lipid Phenotypes Following Roux‐en‐Y Gastric Bypass in Women: An Exploratory Analysis of the SURMetaGIT Study
Source: J Obes. 2026 Aug 12;2026:2335829. doi: 10.1155/jobe/2335829 (PMC13465188; doi:10.1155/jobe/2335829)
Supplement: Supplementary file 1 [file JOBE-2026-2335829-s002.zip › Supp B.docx]

**Supplementary material B: Sphingolipids identified in plasma samples**

| Sphingolipid class | Abbreviation | Cluster 1 | | Cluster 2 | |  |
| --- | --- | --- | --- | --- | --- | --- |
|  |  | P value | Effect size | P value | Effect size |  |
| Ceremide | Cer(d18:1/23:0) | **< .001** | **1.00** | **< .001** | **1.00** | ↓ |
|  | Cer(d18:1/24:0)* | **0.032** | **0.72** | **< .001** | **1.00** | ↓ |
|  | Cer(d18:1/24:1) | **< .001** | **-1.00** | **0.002** | **-0.92** | ↑ |
| Glycosphingolipid | LacCer(d18:1/16:0)** | 0.067 | 0.63 | **0.021** | **0.74** | ↑ |
|  | GlcCer(d18:1/22:0) | 0.465 | 0.27 | 0.064 | 0.61 | ↓ |
|  | GlcCer(d18:1/24:0)* | 0.465 | 0.27 | **0.002** | **0.9** | ↓ |
|  | GlcCer(d18:1/24:1) | **0.014** | **-0.81** | **< .001** | **-1.00** | ↑ |
| Sphingomyelin | SM(d18:1/12:0) | **< .001** | **1.00** | **< .001** | **0.97** | ↓ |
|  | SM(d18:0/14:0) | 0.206 | 0.45 | **0.042** | **0.66** | ↓ |
|  | SM(d18:1/14:0) | **0.002** | **0.96** | **< .001** | **0.97** | ↓ |
|  | SM(d18:2/14:0) | **0.003** | **0.93** | **< .001** | **1.00** | ↓ |
|  | SM(d18:1/15:0) | 0.638 | 0.18 | 0.092 | 0.56 | ↓ |
|  | SM(d18:0/16:0) | 0.206 | 0.45 | 0.151 | 0.48 | ↑ |
|  | SM(d18:1/16:0) | 0.102 | 0.57 | 0.380 | 0.30 | ↑ |
|  | SM(d18:2/16:0) | 0.413 | 0.30 | 0.519 | 0.25 | ↑ |
|  | SM(d18:0/18:0) | **0.026** | **0.71** | 0.204 | 0.43 | ↑ |
|  | SM(d18:1/18:0) | **< .001** | **1.00** | **0.042** | **0.66** | ↑ |
|  | SM(d18:1/18:1) | 0.147 | 0.51 | 0.622 | 0.17 | ↑ |
|  | SM(d18:2/18:1) | 0.206 | 0.45 | 0.970 | 0.02 | ↑ |
|  | SM(d18:0/20:0) | 0.700 | 0.15 | 0.110 | 0.53 | ↓ |
|  | SM(d18:1/20:0)* | 0.175 | 0.48 | **< .001** | **0.97** | ↓ |
|  | SM(d18:1/20:1) | **0.032** | **0.72** | **0.007** | **0.84** | ↑ |
|  | SM(d18:1/21:0) | **< .001** | **1.00** | **< .001** | **1.00** | ↓ |
|  | SM(d18:1/22:0)* | 0.102 | 0.57 | **< .001** | **1.00** | ↓ |
|  | SM(d16:1/24:1) | 0.102 | 0.57 | 0.910 | 0.05 | ↑ |
|  | SM(d18:1/22:1) | **0.014** | **0.81** | **< .001** | **1.00** | ↓ |
|  | SM(d18:1/23:0) | **0.002** | **0.96** | **< .001** | **1.00** | ↓ |
|  | SM(d18:1/24:0)* | **< .001** | **1.00** | **< .001** | **1.00** | ↓ |
|  | SM(d18:2/23:0) | **< .001** | **1.00** | **< .001** | **1.00** | ↑ |
|  | SM(d18:2/24:0) | 0.054 | 0.66 | **< .001** | **1.00** | ↓ |
|  | SM(d18:2/24:1) | **< .001** | **1.00** | **< .001** | **1.00** | ↑ |
|  | SM(d18:2/25:0) | **< .001** | **1.00** | **< .001** | **0.97** | ↑ |

* Sphingolipids with statistical significance only in cluster 2. **LacCer(d18:1/16:0) ou Gal-Gal-Cer (d18:1/16:0). Statistically significant differences are expressed in bold numbers. The arrow pointing up (↑) indicates an increase in metabolite abundance, while the arrow pointing down (↓) indicates a decrease. The 2 arrows displaced together represent the changes in clusters 1 and 2, respectively.
