# Supplementary material for: Preoperative Cer(d18:1/24:0) Shows Promise as a Candidate Predictor of Plasma Lipid Phenotypes Following Roux‐en‐Y Gastric Bypass in Women: An Exploratory Analysis of the SURMetaGIT Study
Source: J Obes. 2026 Aug 12;2026:2335829. doi: 10.1155/jobe/2335829 (PMC13465188; doi:10.1155/jobe/2335829)
Supplement: Supplementary file 1 [file JOBE-2026-2335829-s002.zip › Supp C.docx]

**Supplementary material C: Adjusted binomial logistic regression model for confounding variables**

a)

b)

c)

a) Adjusted binomial logistic regression model for T2DM remission: group 1: remission at 3 months post-surgery; group 2: not achieved remission at 3 months post-surgery; b) Adjusted binomial logistic regression model for lipid-lowering therapy before surgery; c) Adjusted binomial logistic regression model for cholecystectomy. CI: Confidence interval; OR: Odds ratio.
