# Supplementary material for: Preoperative Cer(d18:1/24:0) Shows Promise as a Candidate Predictor of Plasma Lipid Phenotypes Following Roux‐en‐Y Gastric Bypass in Women: An Exploratory Analysis of the SURMetaGIT Study
Source: J Obes. 2026 Aug 12;2026:2335829. doi: 10.1155/jobe/2335829 (PMC13465188; doi:10.1155/jobe/2335829)
Supplement: Supplementary file 1 [file JOBE-2026-2335829-s002.zip › Supp D.docx]

**Supplementary material D: Linear regression of discriminative sphingolipids - continue**

|  | R2 | aR2 | F | p |
| --- | --- | --- | --- | --- |
|  | LacCer(d18:1/16:0) | | | |
| Total Cholesterol mg/dL | 0.002 | -0.04 | 0.04 | 0.88 |
| HDL-c mg/dL | 0.19 | **0.16** | 5.24 | **0.03** |
| Non-HDL-c mg/dL | 0.03 | -0.006 | 0.86 | 0.36 |
| LDL-c mg/dL | 0.001 | -0.04 | 0.03 | 0.85 |
| VLDL-c mg/ dL | 0.004 | -0.04 | 0.10 | 0.75 |
| Triglycerides | 0.003 | -0.04 | 0.08 | 0.77 |
|  | GlcCer(d18:1/24:0) | | | |
| Total Cholesterol mg/dL | 0.19 | **0.15** | 5.00 | **0.03** |
| HDL-c mg/dL | 0.04 | 0.003 | 1.08 | 0.30 |
| Non-HDL-c mg/dL | 0.14 | 0.10 | 3.59 | 0.07 |
| LDL-c mg/dL | 0.03 | -0.01 | 0.73 | 0.40 |
| VLDL-c mg/ dL | 0.02 | -0.02 | 0.52 | 0.47 |
| Triglycerides | 0.02 | -0.02 | 0.48 | 0.49 |
|  | SM(d18:0/14:0) | | | |
| Total Cholesterol mg/dL | 0.11 | 0.06 | 2.59 | 0.12 |
| HDL-c mg/dL | 0.06 | 0.01 | 1.41 | 0.24 |
| Non-HDL-c mg/dL | 0.06 | 0.02 | 1.53 | 0.22 |
| LDL-c mg/dL | 0.009 | -0.03 | 0.19 | 0.66 |
| VLDL-c mg/ dL | 0.06 | 0.02 | 1.48 | 0.23 |
| Triglycerides | 0.004 | -0.04 | 0.10 | 0.75 |
|  | SM(d18:1/20:0) | | | |
| Total Cholesterol mg/dL | 0.30 | **0.26** | 9.07 | **0.007** |
| HDL-c mg/dL | 0.24 | **0.20** | 6.77 | **0.01** |
| Non-HDL-c mg/dL | 0.16 | 0.12 | 4.13 | 0.05 |
| LDL-c mg/dL | 0.06 | 0.01 | 1.41 | 0.24 |
| VLDL-c mg/ dL | 0.01 | -0.03 | 0.21 | 0.65 |
| Triglycerides | 0.003 | -0.03 | 0.20 | 0.65 |

**Appendix D: Linear regression of discriminative sphingolipids - conclusion**

|  | R2 | aR2 | F | p |
| --- | --- | --- | --- | --- |
|  | SM(d18:1/22:0) | | | |
| Total Cholesterol mg/dL | 0.44 | **0.42** | 17.10 | **<.001** |
| HDL-c mg/dL | 0.12 | 0.08 | 3.08 | 0.09 |
| Non-HDL-c mg/dL | 0.33 | **0.30** | 10.51 | **0.004** |
| LDL-c mg/dL | 0.24 | **0.20** | 6.76 | **0.01** |
| VLDL-c mg/ dL | 0.03 | -0.01 | 0.73 | 0.40 |
| Triglycerides | 0.03 | -0.01 | 0.69 | 0.41 |
|  | SM(d18:1/24:0) | | | |
| Total Cholesterol mg/dL | 0.50 | **0.48** | 21.32 | **<.001** |
| HDL-c mg/dL | 0.08 | 0.04 | 1.93 | 0.17 |
| Non-HDL-c mg/dL | 0.41 | **0.38** | 14.73 | **<.001** |
| LDL-c mg/dL | 0.27 | **0.23** | 7.81 | **0.01** |
| VLDL-c mg/ dL | 0.06 | 0.01 | 1.34 | 0.25 |
| Triglycerides | 0.05 | 0.01 | 1.27 | 0.27 |

HDL-c: high-density lipoprotein; LDL-c: low-density lipoprotein; VLDL: very low-density lipoprotein. Differences with statistical significance are expressed in bold.
