# Supplementary material for: Preoperative Cer(d18:1/24:0) Shows Promise as a Candidate Predictor of Plasma Lipid Phenotypes Following Roux‐en‐Y Gastric Bypass in Women: An Exploratory Analysis of the SURMetaGIT Study
Source: J Obes. 2026 Aug 12;2026:2335829. doi: 10.1155/jobe/2335829 (PMC13465188; doi:10.1155/jobe/2335829)
Supplement: Supplementary file 1 [file JOBE-2026-2335829-s002.zip › Supp E.docx]

**Supplementary material E: Characteristic (ROC) curve of Cer(d18:1/24:0), SM(d18:1/22:0), and SM(d18:1/24:0) to predict post-surgical lipid profile improvement in the pre-operative period**

a)

b)

c)

Area Under the Curve of 0.87 for Cer(d18:1/24:0) (a) and 0.78 for SM(d18:1/22:0) (b) and SM(d18:1/24:0) (c).
